# A monocyte gene expression signature in the early clinical course of Parkinson’s disease

**DOI:** 10.1038/s41598-018-28986-7

**Published:** 2018-07-17

**Authors:** Johannes C. M. Schlachetzki, Iryna Prots, Jenhan Tao, Hyun B. Chun, Kaoru Saijo, David Gosselin, Beate Winner, Christopher K. Glass, Jürgen Winkler

**Affiliations:** 1 Department of Molecular Neurology, University Hospital Erlangen, Friedrich-Alexander Universität (FAU) Erlangen-Nürnberg, 91054 Erlangen, Germany; 2 Department of Cellular and Molecular Medicine, University of California, San Diego at La Jolla, CA 92093-0651 USA; 3 Department of Stem Cell Biology, FAU Erlangen-Nürnberg, 91054 Erlangen, Germany; 4 Department of Molecular and Cell Biology, Helen Wills Neuroscience Institute, University of California, Berkeley, CA 94720-3200 USA; 5 Department of Molecular Medicine, Centre de Recherche du CHU de Québec - Université Laval, Québec, G1V 4G2 Canada

**Keywords:** Innate immunity, Parkinson's disease

## Abstract

Microglia are the main immune cells of the brain and express a large genetic pattern of genes linked to Parkinson’s disease risk alleles. Monocytes like microglia are myeloid-lineage cells, raising the questions of the extent to which they share gene expression with microglia and whether they are already altered early in the clinical course of the disease. To decipher a monocytic gene expression signature in Parkinson’s disease, we performed RNA-seq and applied the two-sample Kolmogorov-Smirnov test to identify differentially expressed genes between controls and patients with Parkinson's disease and changes in gene expression variability and dysregulation. The gene expression profiles of normal human monocytes and microglia showed a plethora of differentially expressed genes. Additionally, we identified a distinct gene expression pattern of monocytes isolated from Parkinson’s disease patients at an early disease stage compared to controls using the Kolmogorov-Smirnov test. Differentially expressed genes included genes involved in immune activation such as *HLA-DQB1, MYD88, REL*, and *TNF-α*. Our data suggest that future studies of distinct leukocyte subsets are warranted to identify possible surrogate biomarkers and may lead to the identification of novel interventions early in the disease course.

## Introduction

Parkinson’s disease (PD) is a chronic progressive neurodegenerative disorder^1^ with a highly variable clinical phenotype and disease course^2^. The pathological hallmark of PD is the presence of α-synuclein-containing intraneuronal aggregates^3^. The precise etiology of sporadic PD is unknown, but gene-environment interactions play a major role^4^. Several autosomal dominant and autosomal recessive genes have been identified to cause familial PD, e.g. *SNCA* coding for the protein α-synuclein and *LRRK2* (leucine-rich repeat kinase 2)^5^. The genetic landscape of PD, however, is complex, and more than 50 genetic risk loci for PD have been identified through genome-wide association studies (GWAS)^6–9^. Analyses of GWAS data showed overrepresentation of pathways involved in cytokine-mediated signaling and regulation of lymphocyte activity^10,11^. These genetic studies indicate that dysregulation of the immune system may impact disease risk, clinical phenotype, and progression of PD. Epidemiological and postmortem studies further support the prominent role of myeloid cells in PD pathogenesis^12,13^. Interestingly, we recently showed that approximately one third of the genes associated with PD risk alleles exhibited preferential expression in microglia^14^.

Myeloid cells constitute a heterogeneous class of innate immune cells and microglia represent the major class of myeloid cells in the central nervous system (CNS)^15^. Microglia are derived from a unique lineage of erythromyeloid precursors in the yolk-sac and fetal liver^16–18^. Microglia are long-living cells that can self renew without any significant contribution from circulating myeloid cells under steady-state conditions^19–21^. Microglia survey the brain parenchyma^22^, initiate the brain immune response^23^, and modulate synapse refinement^24^ as well as neurogenesis^25^.

It is currently not feasible to directly assess the brain immune status and brain myeloid cell gene signature in living PD patients. An important question is whether monocytes, a readily accessible population of myeloid cells of the periphery, may provide insight into the myeloid cell status of PD. Monocytes in contrast to microglia are short-lived cells that are generated throughout life from bone marrow hematopoietic stem cells^26,27^. Monocytes are effector cells of the innate immune system and patrol the bloodstream, phagocytose debris, communicate with local cells, and give rise to macrophages or dendritic cells^28,29^. Recent studies suggested that monocytes may serve as contributing factors to PD pathogenesis. An over-representation of expression quantitative trait loci (eQTL) specific to monocytes were linked to PD^30^. Another study showed that monocytes from PD patients respond more severely to TLR4 activation compared to monocytes derived from controls^31^. Monocytes may, in addition, directly infiltrate the brain and contribute to neurodegeneration and α-synuclein accumulation^32–34^. Knowledge of the similarities and differences in the gene expression patterns of human monocytes and microglia are needed to better interpret these findings, but systematic analysis of human microglia gene expression data has only recently become available^14,35^.

Given the lineage relationships of microglia and monocytes, and mounting evidence that the brain is not a fully immune-privileged organ, we hypothesized that monocytes show a distinct gene expression signature already in the early course of PD. We therefore directly compared human monocyte and microglia transcriptomes and assessed the monocytic transcriptome profile in PD patients with a disease duration of less than three years. These studies establish the relative expression of genes associated with PD risk alleles in monocytes and microglia and provide evidence for a differential gene signature in monocytes of individuals with PD at an early stage of the disease, which could reveal novel intervention targets.

## Results

### Study population

The study cohort consisted of ten male PD patients and ten male controls (Table 1). Average age between both groups was not significantly different, although the mean of the control group (61.8 years) was slightly greater than the mean of the PD group (58.6 years). We included only PD patients with a disease duration of three years or fewer. All together, eight patients were categorized to be in an early stage of the disease (Hoehn and Yahr stages 1 and 2) and two patients were classified as Hoehn and Yahr stage 3, indicating an intermediate stage of the disease. The short disease duration was also reflected by a low motor score on part III of the Unified Parkinson’s Disease Rating Scale (UPDRS-III, 15.7 ± 2.2) and a low levodopa equivalent daily dose (LEDD, 309.5 ± 64.6).

**Table 1:** Study population.

No.	PD group	Control group
	10 males	10 males
Age (years)	58.6 ± 4.1	61.8 ± 3.9
Disease duration (years)	2.2 ± 0.2	
Hoehn and Yahr	1.8 ± 0.3	
Stage 1	n = 4	
Stage 2	n = 4	
Stage 3	n = 2	
UPDRS-III	15.7 ± 2.2	
LEDD (mg/d)	309.5 ± 64.6	

Values are depicted as mean ± S.E.M.

UPDRS-III = Unified Parkinson’s Disease Rating Scale Part III; LEDD = levodopa equivalent daily dose.

### Gene expression signature of human monocytes

**Figure 1:**
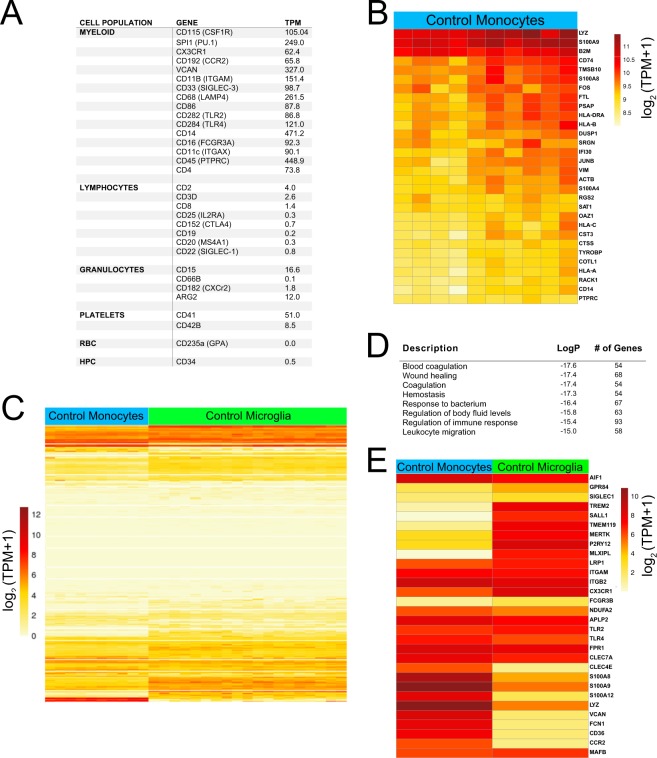
Comparison of control human monocyte and microglia transcriptomes. (**A**) Gene expression of selected genes associated with various immune cell types in monocytes isolated from healthy controls revealed by RNA-seq. (**B**) Gene expression of the 30 most highly expressed genes in human control monocytes from individual control patients. (**C**) Heat map of mRNA expression values determined in monocytes from 10 controls and in microglia from 19 controls^14^. 3054 genes exhibited > two-fold higher average expression in monocytes compared to microglia. (**D**) Gene Ontology enrichment analysis of the top 1000 genes most preferentially expressed in monocytes supports their role as effector cells in immune response. (**E**) Heat map of mean mRNA expression levels of selected monocyte and microglia genes. RBC - red blood cells; HPC - hematopoietic precursor cells; CD - cluster of differentiation; TPM - transcripts per kilobase million.

We first determined the transcriptome profile of human monocytes isolated from 10 male control individuals (Fig. 1). To this aim, total monocytes were isolated and enriched from peripheral blood mononuclear cells (PBMCs) by density-gradient centrifugation and further purified by untouched magnetic bead isolation. By choosing this strategy, we excluded non-monocyte cell subpopulations such as T cells, NK cells, B cells, dendritic cells, eosinophils, and basophils without directly labeling and potentially activating monocytes. Gene expression signature of human control monocytes was analyzed using edgeR^36^. Monocytic transcriptomic data indicated low to absent expression levels of non-monocyte cell markers (Fig. 1A). Relative expression values for the 30 most highly expressed genes in monocytes across each of control individual are illustrated in Fig. 1B, indicating substantial individual variation for some transcripts. This analysis showed that human monocytes expressed very high levels of numerous genes associated with antigen processing and presentation (*IFI30, HLA-DRA, HLA-A, HLA-B, HLA-C*) and regulation of the innate immune response (*LYZ, S100A9, ACTB, S100A8, S100A4, TYROBP, CD14*). In addition, *FOS* and *JUNB* as members of the AP-1 transcription factor family were highly expressed in monocytes.

Monocytes and microglia are both classified as myeloid cells, but reside in very distinct compartments. To test the impact of the respective niche, we compared the gene expression pattern of monocytes and microglia from control individuals. We applied a cutoff of two-fold change in expression in monocytes relative to microglia at a false discovery rate (FDR) of 0.05. A total of 3054 genes were up-regulated and 3461 genes were down-regulated in monocytes compared to microglia (Fig. 1C). Gene enrichment analysis for the top 1000 genes most preferentially expressed in monocytes compared to microglia showed a strong association to processes involved in blood coagulation and wound healing, regulation of immune response, including leukocyte migration (Fig. 1D). Gene ontology enrichment analysis for the top 1000 genes most preferentially expressed in microglia compared to monocytes included synaptic transmission (logP −8.4) and developmental growth involved in morphogenesis (logP −7.2). Genes that were highly expressed in monocytes compared to microglia included *S100A8, S100A9, S100A12, LYZ, CCR2, CD36, FCN1*, and *VCAN* (Fig. 1E). In contrast, *TREM2, SALL1, TMEM119, MERTK, MLXIPL*, and *CX3CR1* were increased in microglia compared to monocytes.

### Routine peripheral laboratory tests and distribution of monocyte subpopulation

**Figure 2:**
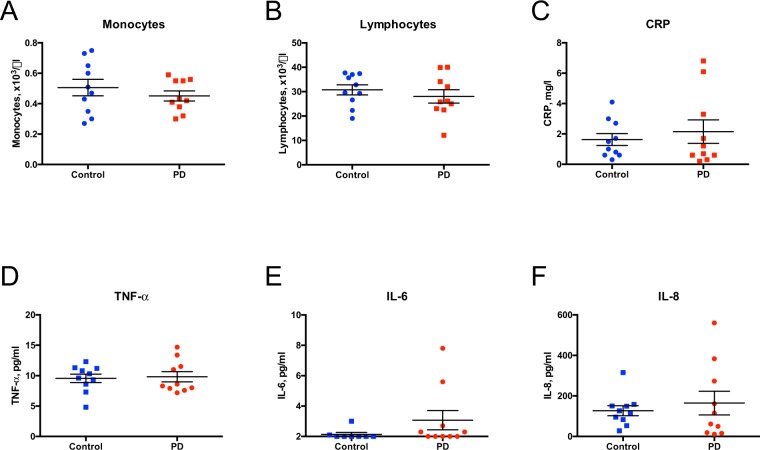
Results of the routine laboratory tests of peripheral blood samples. The routine laboratory tests for monocytes (**A**) and leukocytes (**B**) were not changed between PD and control peripheral blood samples. Whereas there were no statistically significant differences in CRP (**C**), TNF- α (**D**), IL-6 (**E**), and IL-8 (**F**) levels, variability was increased in the PD cohort. Results are depicted as mean ± SEM. To test for significant differences, Student t-test was performed.

**Figure 3:**
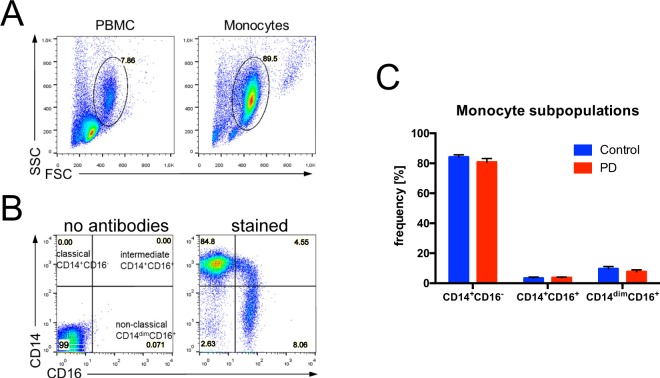
Flow cytometry data analysis of human monocyte subsets in early-course PD and control individuals. (**A**) After density gradient, PBMCs were visualized using Forward Scatter (FSC) vs. Side Scatter (SSC). Monocytes were enriched from PBMCs by negative isolation using magnetic beads. More than 90% of isolated monocytes were viable as determined by gating in FSC vs. SSC (**B**) Gating strategy for monocyte characterization and separation in classical CD14^+^CD16^−^, intermediate CD14^+^CD16^+^, and non-classical CD14^dim^CD16^+^ subpopulations. (**C**) Quantification of monocyte subpopulation based on CD14 and CD16 expression revealed no significant differences between PD and control individuals. Mean ± SD, Student t-test, p > 0.05.

Next, we compared standard laboratory parameters and distribution of monocyte subpopulations within the present cohort (Fig. 2). Total monocyte and lymphocyte numbers, as well as C-reactive protein (CRP) and tumor necrosis factor (TNF)-α, interleukin (IL)-6, IL-8 cytokine levels in the peripheral blood were unchanged between early course PD patients and controls (Student’s unpaired t-test, p-value > 0.05; Fig. 2). After untouched selection with magnetic beads, flow cytometry analysis based on CD14 and CD16 expression (Fig. 3A,B) revealed no significant differences in the distribution of classical (CD14^+^CD16^−^), intermediate (CD14^+^CD16^+^), and non-classical (CD14^dim^CD16^+^) monocyte subpopulations between PD and controls (Fig. 3C).

### Comparison of transcriptome profiles of monocytes between PD and control individuals

**Figure 4:**
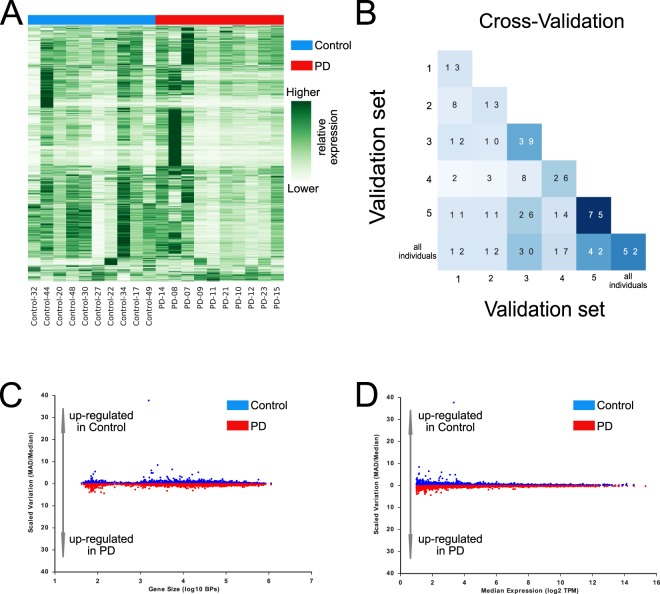
Comparison of early-course PD and control monocyte transcriptomes. (**A**) Heat map of mRNA expression values determined in monocytes of 10 controls and 10 early course PD individuals. (**B**) Overlap matrix of differentially expressed genes calculated by edgeR for 5 cross validation sets formed by randomly excluding two PD patients and two control individuals as well as the entire cohort. (**C**) Gene size versus the variability of gene expression. Gene size is quantified as the log10 transform of the size of each gene transcript in basepairs (BPs). Variability of gene expression is measured using the median absolute deviation (MAD) scaled by the median expression value within each group. (**D**) Expression level versus the variability of gene expression. Gene expression is quantified as the log2 transform of the median expression value within each group measured in TPM. Variability of gene expression is measured using the MAD scaled by the median expression value within each group.

Next, we asked whether monocytes isolated from individuals in the early clinical stage of PD show a distinct disease-associated gene expression signature compared to control subjects. Hierarchical clustering of the expression of genes expressed at 10 transcripts per kilobase million (TPM) or greater in at least one sample in monocytes from PD patients and control individuals is illustrated in Fig. 4A (expression values are normalized to fall between 0 and 1). Notably, the expression level of each gene varied considerably both across all individuals and within the control and PD groups. Next, we sought to identify differentially expressed genes in monocytes isolated from PD and control patients using edgeR. EdgeR is a conventional RNA-seq analysis software tool that models expected noise and variability in RNA-seq replicate experiments in order to identify differentially expressed genes. With a fold-change of two as a cut-off and FDR set at 0.05, edgeR identified a total of 52 genes (lower right corner, Fig. 4B). Given the considerable variability in gene expression we had observed within each group of patients, we considered the robustness of the edgeR to outlier gene expression values. To assess the robustness of the edgeR results, we created five cross validation sets by randomly excluding two PD patients and two controls from each cross validation set, and identified differentially expressed genes for each cross validation set using edgeR by selecting genes that had a FDR less than 0.05. The heat map depicted in Fig. 4B shows how many differentially expressed genes reported by edgeR overlap between cross validation sets and the entire cohort. For example, of the 13 differentially expressed genes found for cross validation set 1 and the 39 for cross validation set 3, we observed an overlap of 12 genes. Thus, edgeR results are not robust when examined under cross validation conditions.

While the poor overlap between differentially expressed genes discovered in each of the cross-validation sets could be potentially attributed to the limited sample size, we noted that edgeR was designed to function in scenarios where there are as few as one or two replicate experiments. Thus, we considered the alternative hypothesis that the poor overlap between the cross-validation sets may be due to inherent variation in gene expression due to natural genetic variation. At first, we quantified the variability in expression for each gene using the Median Absolute Deviation (MAD), which is the absolute value of the median difference between each expression value and the median expression value. We scaled the MAD by the median expression value to account for differences in the scale of expression of different genes (some genes are expressed at 1000s of TPM whereas others are expressed at 10s of TPM). We observed that the variability of expression for each gene could vary between the PD and control groups and that these differences did not stratify according to gene size (Fig. 4C). We also observed that genes that demonstrated differential variability tended to be moderately expressed genes (Fig. 4D). These observations led us to develop an analysis pipeline to deconvolute the change in gene expression levels and the change in the variability of gene expression.

### Extended analyses of RNA-seq data

**Figure 5:**
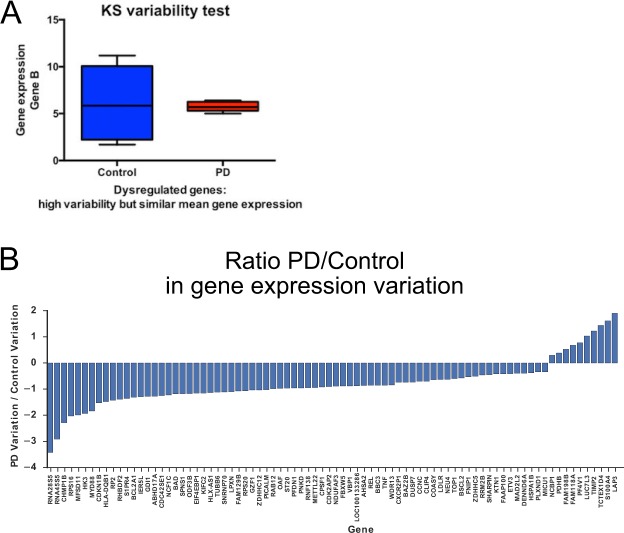
Gene expression in early-course PD is more heterogeneous compared to control monocytes. (**A**) Principle of RNA-seq data analysis with two-sample KS variability test to analyze differences in gene expression variability. (**B**) Bar graph depicting dysregulated genes according to their heterogeneity in gene expression as determined by two-sample KS test. Shown here is the ratio of gene expression variation between PD and control monocytes.

We asked whether gene expression variability is altered in PD monocytes compared to controls (Fig. 5A). Given our limited sample size, we implemented a nonparametric statistical framework to test: 1) differences in the variability of gene expression in the two groups and 2) differences in the expression level of each gene. Nonparametric statistical tests tend to have more robust behavior when sample size is limited. We applied the Kolmogorov-Smirnov (KS) test in both scenarios. The KS test is a nonparametric test that assesses whether two sets of continuous values are drawn from the same distribution. We tested for differences in gene expression level by directly applying the KS test to the expression values of each gene from the PD and control group. To test for differences in variability, we first quantified variability of each gene by calculating the absolute difference between each expression value and the median expression value within each group, and then applied the KS test to these absolute difference values. The genes illustrated in Fig. 5B pass a KS test for unequal variance (p < 0.01), but not for differential gene expression level. Our results reveal dramatic differences in gene expression variability; 73 genes exhibited greater variation in control monocytes than in PD monocytes, whereas 9 genes showed greater variation in PD monocytes (Supplementary Table 1; snoRNAs and miRNAs were excluded). Genes that showed decreased monocytic gene expression variability in PD patients compared to controls included in inflammatory pathway such as *HLA-DQB1, MYD88, REL*, and *TNF-α as well as CHMP1B*, which is involved in the formation of endocytic multi-vesicular bodies.

**Figure 6:**
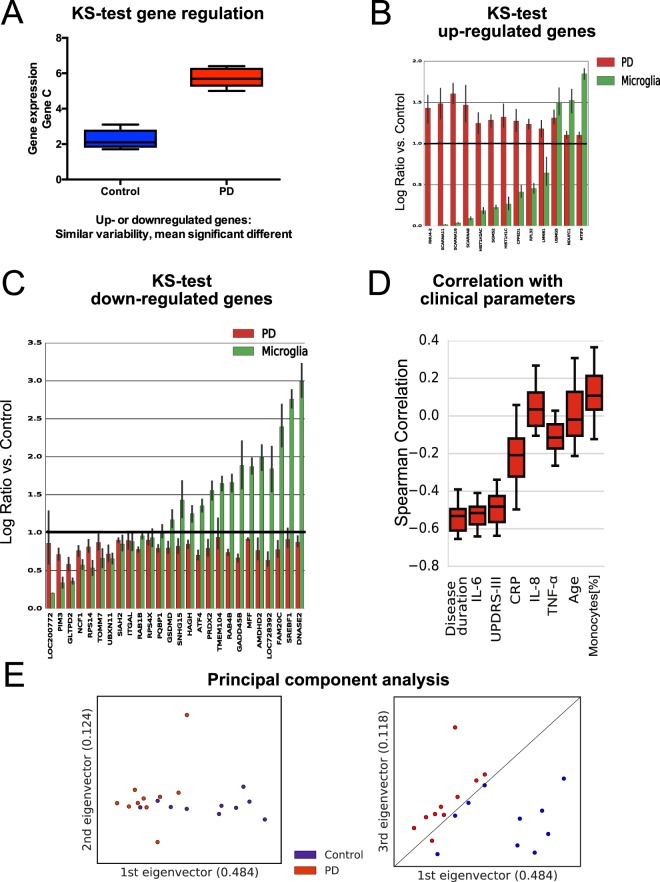
Differentially expressed genes in monocytes isolated from early-course PD patients. (**A**) Scheme showing genes with similar gene expression variability but significantly altered mean gene expression using the KS expression test. (**B, C**) Log ratio of genes regulated in PD monocytes and microglia vs. control monocytes. Upregulated genes in PD monocytes compared to controls using the KS expression test is depicted in (**B**), down-regulated genes in PD monocytes compared to controls in (**C**). (**D**) Correlation analysis of differentially expressed genes identified by the KS-test and clinical parameters. (**E**) Principal component analysis (PCA) of differentially expressed genes as determined by the two-sample KS-expression test separates PD patients from control individuals.

We then analyzed genes that showed similar gene expression variability but statistically different median gene expression (p < 0.01) Fig. 6A; Supplementary Table 2). 13 genes were up-regulated (Fig. 6B) and 26 down-regulated (Fig. 6C) in PD monocytes (after miRNAs and snoRNAs were excluded). These differentially expressed genes were robust in a cross validation experiment. Across five cross-validation where 2 PD and 2 control patients were randomly excluded, each of the 39 differentially expressed genes identified using the KS test had a maximum p-value less than 0.05. Among the down-regulated genes using the KS test were the transcription factors *ATF4 and SREBF1* and genes involved in mitophagy like *TOMM7*. Interestingly, 10 out of the 13 up-regulated genes in PD monocytes were lower expressed in human microglia, suggesting that there may be a monocyte specific PD-specific transcriptional signature (Table 2).

**Table 2:** Chi-square test for dysregulated genes in human monocytes and microglia.

	PD *vs* Control monocytes			χ^2^ = 4.18 P = 0.041
	Up-regulated	Down-regulated		
18	3	15	Up	Control monocytes *vs* Microglia
21	10	11	Down	
	13	26		

Next, we asked whether the genes identified in the transcriptional analysis relate to clinical parameters. To address this question, we determined the Spearman correlation of each of the 26 down-regulated PD signature genes that passed the KS expression test and the parameters: age, disease duration, TNF-α, IL6, IL-8, CRP, UPDRS-III score, and monocytes. Disease duration, UPDRS-III score, and IL-6 highly correlated with each of the down-regulated genes that passed the KS expression test for differential expression (Fig. 6D). However, the clinical parameter age did not correlate with the PD gene expression signature.

Next, we performed principal component analysis (PCA) to assess whether gene expression patterns can be used to segregate PD monocytes and control monocytes. PCA on the 39 differentially expressed genes reveal that these genes form a signature that distinguishes early-stage PD patients from controls (Fig. 6E).

Together, our analysis determined an early-stage PD transcriptome signature of peripheral monocytes that is characterized by dysregulated genes involving inflammatory pathways. Moreover, although the number of differentially expressed genes between PD and controls is low, variation analysis and PCA confirm a disease-specific pattern in PD monocytes that correlated with disease severity.

### Monocyte gene expression of genes containing PD risk alleles

**Figure 7:**
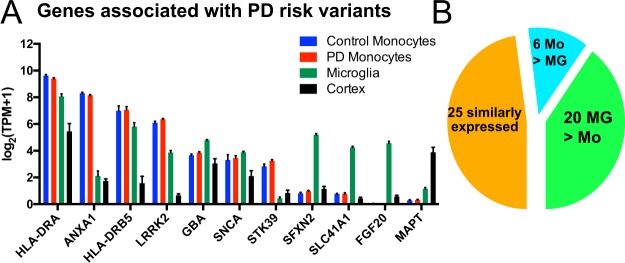
Relative expression of genes associated with PD risk genes. (**A**) Bar graph showing the relative mean and 95% Confidence interval (CI) of the log_2_(TPM + 1) values of 17 genes associated with PD risk in human monocytes from controls (blue), PD (red), human microglia (green), and human cortical bulk tissue (black). Only genes with TPM > 2 are shown. (**B**) Pie chart depicting the number of genes with at least two-fold higher expression in either human monocytes (Mo, blue), microglia (MG, green), or similarly expressed in both cell types (orange).

Characterization of the monocyte transcriptome, in conjunction with microglial gene expression profiling, enabled the evaluation of the relative expression levels of genes associated with PD risk. We took advantage of a GWAS database^9^ to identify PD risk alleles. Taking the nearest gene approach, we compared the expression levels of 51 genes associated with an increased risk of PD in monocytes from PD and control individuals. The highest expressed genes in monocytes were *HLA-DRA*, *ANXA1*, *HLA-DRB5*, and *LRRK2* (Fig. 7A). Genes that were expressed at low levels in monocytes included *DLG2, SYT12, SH3GL2, FGF20, SYT4, STX1B, MAPT, SLC41A1, SFXN2, UNC13B, GPNMB*, and *HIP1R*.

Finally, we compared the mean expression level of these genes in control monocytes to human microglia and human bulk cortical brain tissue (Fig. 7A)^14^. LRRK2 mRNA was preferentially expressed in human monocytes and microglia, whereas *MAPT* mRNA expression was mainly found in bulk tissue from human cortex. Interestingly, only 6 genes (12%) were expressed two-fold higher in monocytes compared to microglia, most notably LRRK2 (Fig. 7B). Twenty genes (49%) were higher expressed in microglia than monocytes, e.g. *FGF2*0, *GPNMB*, and *SFXN2*. About half of the genes were similarly expressed in both cell types.

## Discussion

There is an increasing effort to leverage peripheral blood cells to identify blood-based biomarkers in PD^37–39^. The present study provides evidence for a distinct transcriptomic signature in monocytes from PD patients in their early disease course. While acknowledging a significant, although subtle, monocytic transcriptomic signature in PD, we show that the innate peripheral immune system is altered early in the clinical course of patients with PD. Indeed, we found a distinct signature that separates PD and controls with respect to clinical score and disease duration.

It is interesting to note, that when comparing gene expression in our control monocyte cohort with a human microglia cohort reported previously^14^, a large number of genes is expressed at significantly higher levels in monocytes. Among those genes, biological processes involved in leukocyte migration and regulation of immune responses are enriched, pointing a connection between innate and adaptive immune responses. This is indicative of the potent role of monocytes as effector cells in immune response and leads the way towards studying a cell type that can be approached easily at different disease stages in patients to decipher a temporal course of neuroinflammation. Taken together, we now know which genes associated with PD risk alleles are similarly expressed in monocytes and microglia. This information provides the basis for investigating functional roles of the corresponding genes in monocytes and whether the expression levels of these genes in monocytes is predictive of their expression in the brain.

The PCA of the RNA-seq data based on the expression levels of down-regulated genes in PD monocytes shows a signature that distinguishes early-stage PD patients from controls. This finding extends a previous gene expression study in peripheral monocytes of PD patients of mixed disease and duration^31^. Of note, the comparison of all PD disease stages in the previous study versus distinct disease stages changes the differential gene expression pattern, suggesting that gene expression profile in monocytes might change during disease progression. When comparing our study and the study by Grozdanov *et al*., there was no overlap of determined differentially expressed genes, most likely due to the defined early disease stage in the present patient cohort^31^. Notably, no excess expression of cytokines TNF-α and IL-6 was detected previously in unstimulated PD monocytes compared to controls^31^, which is in accordance with our findings.

Recent studies in amyotrophic lateral sclerosis (ALS) identified dysregulation of inflammatory and immune-related genes in ALS monocytes^40^ and a unique inflammation-related gene expression profile with more than 200 differentially expressed genes in unstimulated monocytes from patients compared to controls^41^. Notably, the latter study showed that the number of proinflammatory differentially expressed genes correlated with the course of disease progression. Significant transcriptional differences indicative of a proinflammatory phenotype were also described in Huntington’s disease (HD) monocytes in an unstimulated state^42^. Taken together, these data point towards unifying proinflammatory signatures of peripheral myeloid cells and rendering those an important cell population for understanding disease modification and progression in neurodegenerative diseases.

Using the KS expression test, we identified differentially expressed genes in PD monocytes that, in contrast genes identified by edgeR, robustly separated the gene expression signature in PD monocytes from controls. For example, *ATF4*, a member of the AP-1 transcription factor family, was down-regulated in PD monocytes. Altered levels of ATF4 has been attributed to cell death in PD^43,44^. Other genes that were down-regulated involved mitochondrial function, in particular mitochondrial protein synthesis (*MTIF3*)^45^, mitophagy (*TOMM7, SREBF1)*^46–48^ and stress defense (*PRDX2*)^49^.

Gene expression variability has been associated with human genetic variation and human disease and is highly influenced by genetic and environmental factors^50–52^. Genes with low variance could indicate that these genes themselves are highly constrained. Here we show differential gene expression variability in PD monocytes compared to control monocytes. Differences in gene expression variability may point to disruptions in transcriptional regulatory mechanisms, which may translate to different distinct PD phenotypes via dysfunction of associated pathways. This was also shown by a recent study that showed altered gene expression variability in neural stem cells derived from PD patients^53^.

When analyzing expression profile of PD risk genes, no significant differences were detected between PD and control monocyte populations. This might be either explained by the early disease stage, when expression of the PD risk genes is not altered yet. Alternatively, it might suggest either the low penetrance of the identified risk alleles, or a role for allele specific gene expression. The wide array in the diversity of PD pathogenesis could potentially be explained by the hypothetical presences of many low penetrance risk alleles that each contribute to the pathogenesis of PD in varied ways. However, given the size of our study population and the lack of genotype data in this study, we cannot make any conclusive statements about risk allele penetrance. We also cannot draw conclusions about whether an individual that inherits a risk allele on both chromosomes are at greater risk of PD than an individual that inherits a risk allele on just one chromosome. Given the complexity of PD pathogenesis, we believe that genotype data may be an important resource that must be paired transcriptome profiling in future studies of PD.

Expression of the gene encoding α-synuclein (*SNCA*), was not altered in our PD cohort and control monocytes and matching the expression level of control microglia. On the other side, increased protein levels of α-synuclein were demonstrated in monocytes from α-synuclein overexpressing mice and PD patients^54^, indicative of differences in post-translational regulation of α-synuclein in monocytes. It is interesting to note that phagocytosis of α-synuclein is impaired in aging and PD monocytes^55^, and in microglia of lymphocyte-depleted α-synuclein over-expressing mice^56^. In light of α-synuclein as an antigenic epitope in T cells, which drives the adaptive immune system, and specifically helper and cytotoxic T cell responses in PD patients^57^, an increase of α-synuclein in the innate peripheral immune cells is a potential proinflammatory stimulus.

Analyses for numbers of monocytes and lymphocytes, cytokine and CRP serum levels did not reveal significant differences in our study, but various altered levels of peripheral cytokines and chemokines levels were reported in various PD cohorts^58,59^. These values are most likely not specific enough to reliably detect a chronic subtle inflammatory process in PD, but require meta-analyses.

In future studies, additional attention should be directed to monocyte subpopulations with diverse functions, since they may help to define specific inflammatory signatures in neurodegeneration. The different subpopulations of monocytes were not altered in terms of frequencies in our study. However, monocyte subsets also revealed differential clusters by single cell RNA-seq^60^ rather than a homogeneous population^61–63^.

Taken together, our findings indicate a disease-specific gene expression profile of peripheral monocytes in early course of PD that correlates with disease severity. Thus, our study significantly adds to the knowledge of the gene expression signatures of monocytes in PD and encourages further investigations on monocyte function in PD. Understanding the monocytic transcriptional profile and function in PD may offer new promising targets for disease-modifying therapies and serve as potential clinical biomarker for disease progression. Our results are consistent with the possibility that gene-environment interactions alter responses of myeloid lineage cells and thus contribute to both, PD pathogenesis and its broad range disease progression.

## Material and Methods

### Study population

All participants were recruited from the movement disorder outpatient center of the University Hospital Erlangen, Germany. The study was approved by the ethics committee of the FAU Erlangen-Nürnberg and written informed consents were provided from all participants prior to sample collection. All patients were interviewed and examined by a board-certified neurologist. Patients were diagnosed with PD according to the National Institute of Neurological Disorders and Stroke [NINDS] diagnostic criteria for PD^64^. Overall disease severity was assessed by the Hoehn and Yahr PD staging scheme (range 1–5)^65^. Motor impairment was assessed using the UPDRS motor score part III rating^66^. Type and dose of dopaminergic medication was compared using LEDD calculated as previously published^67^. Only PD patients with a disease duration of three or less years and in an early to moderate stage of the disease (Hoehn and Yahr disease stage 1 to 3) were eligible to participate. We only considered sporadic PD cases without any clear attribution to a specific genetic or environmental cause. We recruited 10 age- and sex-matched healthy control individuals without current signs or diagnoses of neurological disorders.

The following exclusion criteria were applied to all participants: history of infectious disease in the last 3 months prior to participation, autoimmune diseases, diagnosis of malignancies, signs of neurocognitive disorders, being unable to give informed consent, individuals taking antibiotics and/or steroids.

All experiments were performed in accordance with relevant guidelines and regulations.

### Immunological routine laboratory diagnostics

Routine laboratory tests including white blood cell count and serum levels for CRP, tumor necrosis factor (TNF) -α, interleukin (IL)-6, and IL-8 were performed in peripheral blood samples by the Department of Internal Medicine 3 of the University Hospital Erlangen.

### Monocyte isolation from whole blood samples

Blood collection into heparin-coated vials was performed between 8 and 9 am from fasting individuals. The blood was immediately processed for routine laboratory tests and monocyte isolation. PBMCs were purified from whole blood using a density gradient (Lymphoprep, Axis-Shield, Oslo, Norway) centrifugation. Monocytes were isolated from PBMCs by negative selection with magnetic beads using Pan Monocyte Isolation Kit according to the manufacturer’s instructions (Miltenyi Biotec, Bergisch Gladbach, Germany). Untouched monocytes were either immediately subjected to flow cytometry or pelleted and lysed in RLT buffer (Qiagen, Hilden, Germany), and then stored at −80 °C until RNA isolation.

### Flow cytometry

PBMCs and monocytes were washed and stained with the fluorescently-labeled antibodies to cell surface markers CD14 (APC-labeled) and CD16 (FITC-labeled; both antibodies BD Biosciences, San Jose, CA) by incubation for 15 minutes (min) at +4 °C in the presence of 20 μl Fc Receptor (FcR) Blocking Reagent per 10^7^ total cells (Miltenyi Biotec). Flow cytometry analysis of monocyte subset composition was performed on a BD FACS Aria2 based on CD14 and CD16 expression collecting at least 10,000 events^68^. Flow cytometry data were evaluated using FlowJo software (FlowJo, LLC, Ashland, Oregon).

### RNA isolation and RNA-sequencing (RNA-seq) library preparation

Total RNA was purified using the RNeasy Mini Kit according to the manufacturer’s instructions (Qiagen). Poly(A) selection of RNA was performed as previously described^69^. Briefly, RNA samples were incubated with Oligo d(T) Magnetic Beads (NEB, Ipswich, MA) and poly(A) enriched RNA was collected. To fragment poly(A) RNA, samples were incubated at 94 °C for 9 min on beads. First-strand synthesis and second-strand synthesis as well as well as blunting, A-tailing, and adapter ligation was done using barcoded adapters (NextFlex, Bioo Scientific) as previously described^69^. Libraries were PCR-amplified for 12–15 cycles and size selected for fragments (200–350 bp) by gel extraction (10% TBE gels, Life Technologies EC62752BOX). RNA-seq libraries were single-end sequenced for 51 cycles on an Illumina HiSeq 2500 (Illumina, San Diego, CA) according to manufacturer’s instruction.

### Differential gene expression and variability analyses

FASTQ files from sequencing experiments were mapped to the UCSC genome build hg38. STAR, with default parameters, was used to map RNA-seq experiments^70^. The “analyzeRepeats” function of HOMER was used to generate a table of read counts for each experiment using the parameters “-raw -count exons -strand both -condenseGenes” and a table of TPM values using “-tpm -normMatrix 1e7 -count exons -strand both -condenseGenes”^71^. The base-2 logarithm of the TPM values was taken after adding a pseudocount of 1 TPM to each gene. Un-normalized reads within each gene body were used as input for edgeR^36^. Significance between monocytes from PD and control groups was assessed at a FDR of 0.05 using the Benjamini-Hochberg method and an effect size cutoff of twofold change in expression. The robustness of the edgeR results was assessed using stratified fivefold cross validation; two control and two PD patients were randomly excluded from and the outputs from edgeR for each cross-validation set were compared.

To define the change in the variability of gene expression separately from the change in gene expression, we leveraged the two sample KS test. First, we tested the change in the gene expression level between controls and PD patients for each gene (KS expression level test). Second, we tested the change in the gene expression variability for each gene by determining the absolute distance between the median gene expression level within each cohort and the gene expression level of each individual and the absolute gene expression distance between both cohorts (KS variability test). To ensure that genes identified using these tests were robustly expressed in at least one sample, we required that each gene must be expressed at 10 TPM or greater in one sample. Using both KS tests, we defined (i) differentially expressed genes with low variability as genes that passed the KS expression test (p < 0.01), but not the KS variability test (p > 0.01), and (ii) dysregulated genes as genes that were not differentially expressed (p > 0.01) but showed high variability in gene expression (p < 0.01). Statistical tests were performed using the scipy Python module. The principal component analysis was performed using the sklearn Python module after normalizing the expression values of each gene such that the mean value for each gene is zero and the variance is one (standardization).

To compare human monocyte gene expression signature with human microglia signature and bulk cortical brain tissue of control individuals we used our previously published datasets on human microglia isolated from surgically resected tissue^14^ (GEO repository accession number GSE89960). Genes were considered differentially expressed between human monocytes and microglia if they exhibited a two-fold difference at an FDR of 0.05. Heat maps were generated with the pheatmap package in R.

### Analysis of association between PD risk alleles and gene expression

To study possible associations between gene expression in monocytes and risk genes implicated in PD, we used the GWAS catalog^9^ as previously published^14^. Genes in the vicinity of disease-associated single-nucleotide polymorphism (SNPs) were assigned by the GWAS catalog, and these lists were intersected with gene symbols of our data. Pie charts were generated to display the ratio of all genes that were expressed more than two-fold higher in one condition compared to the other.

### Analysis of clinical attributes

Demographic characteristics including age, disease duration and the clinical rating scales, are given as mean ± standard error of mean (SEM). Potential differences in age between PD patients and controls were analyzed using a two-sample t-test. A significance level of p < 0.05 was set to be statistically significant.

## Electronic supplementary material

Supplementary Table 1

Supplementary Table 2
