# Author Correction: A monocyte gene expression signature in the early clinical course of Parkinson’s disease

**DOI:** 10.1038/s41598-020-62928-6

**Published:** 2020-04-07

**Authors:** Johannes C. M. Schlachetzki, Iryna Prots, Jenhan Tao, Hyun B. Chun, Kaoru Saijo, David Gosselin, Beate Winner, Christopher K. Glass, Jürgen Winkler

**Affiliations:** 1 Department of Molecular Neurology, University Hospital Erlangen, Friedrich-Alexander Universität (FAU) Erlangen-Nürnberg, 91054 Erlangen, Germany; 2 Department of Cellular and Molecular Medicine, University of California, San Diego at La Jolla, 92093-0651 CA USA; 3 Department of Stem Cell Biology, FAU Erlangen-Nürnberg, 91054 Erlangen, Germany; 4 Department of Molecular and Cell Biology, Helen Wills Neuroscience Institute, University of California, Berkeley, 94720-3200 CA USA; 5 Department of Molecular Medicine, Centre de Recherche du CHU de Québec - Université Laval, Québec, G1V 4G2 Canada

Correction to: *Scientific Reports* 10.1038/s41598-018-28986-7, published online 17 July 2018

This Article contains an error in the Acknowledgements section, where

“Sequencing datasets generated for this study have been submitted to Gene Expression Omnibus (GEO) repository (accession number GSE888888).”

should read:

“Sequencing datasets generated for this study have been submitted to Gene Expression Omnibus (GEO) repository (accession number GSE88888).”

